# Influences of Maternal, Child, and Household Factors on Diarrhea Management in Ecuador

**DOI:** 10.3390/children12040473

**Published:** 2025-04-07

**Authors:** Karla Vargas-Gaibor, Kevin Rendón-Viteri, Geovanny Alvarado-Villa, Marco Faytong-Haro

**Affiliations:** Facultad de Ciencias de la Salud, Universidad Espiritu Santo, Samborondon 0901-952, Ecuador; violetavargas@uees.edu.ec (K.V.-G.); kevinrendon@uees.edu.ec (K.R.-V.); galvarado@uees.edu.ec (G.A.-V.)

**Keywords:** acute diarrhea, child healthcare, socioeconomic conditions, maternal and child hygiene habits, child morbidity

## Abstract

Background: Acute diarrheal disease remains a leading cause of childhood morbidity and mortality, particularly among children under five. Despite being preventable and treatable, cultural, socioeconomic, and familial factors influence home management. This study examined how these factors shape childhood diarrhea care in Ecuador. Objective: To analyze maternal, child, and household characteristics associated with diarrhea management in children under five years of age using data from the 2018 Ecuadorian National Health and Nutrition Survey (ENSANUT). Methods: This cross-sectional study applied logistic regression models to assess the influence of various factors on diarrhea management practices. Results: Maternal education, ethnicity, child’s age, household size, and urban or rural residence significantly influenced diarrhea management. Households with 4–6 persons (OR = 1.584, *p* < 0.05) and 7–9 persons (OR = 2.006, *p* < 0.05) had higher odds of receiving medical care. However, the child’s sex, birth order, maternal marital status, and socioeconomic status were not significant predictors. Conclusions: Although most children received some form of healthcare, disparities persisted, particularly in education level, ethnicity, and rural residence. These findings highlight the need for targeted maternal health literacy programs, culturally tailored interventions, and improved water-security initiatives to enhance diarrhea management and reduce inequities in care.

## 1. Introduction

Diarrhea, defined by the World Health Organization (WHO) as three or more loose or liquid stools per day, remains a major cause of childhood morbidity and mortality worldwide [1,2,3]. Despite being preventable and treatable, it accounts for approximately 443,832 deaths among children under five annually and remains a leading cause of mortality in this age group [3]. Globally, there are nearly 1.7 billion episodes of childhood diarrhea each year, with a higher incidence in low- and middle-income countries [3,4]. Risk factors include poor sanitation, lack of access to clean water, inadequate hygiene, and limited medical care [5].

As primary caregivers, mothers play a critical role in managing childhood diarrhea [4]. The Integrated Management of Childhood Illness (IMCI) guidelines recommend Oral Rehydration Salts (ORS) and continued feeding, yet adherence remains insufficient due to gaps in maternal knowledge and traditional beliefs [6,7]. Studies from Turkey, Ethiopia, and Burundi have highlighted that maternal education, socioeconomic status, and hygiene awareness influence diarrhea management [1,4]. However, research in Ecuador remains scarce, with prior studies focusing on antibiotic prescription patterns and economic costs rather than on maternal and household determinants of care [8,9,10,11,12].

Childhood diarrhea is a significant public health concern in Ecuador, particularly among vulnerable populations. The 2018 National Health and Nutrition Survey (ENSANUT) reported that 23.7% of children under five suffered from chronic malnutrition, a condition closely linked to diarrheal disease and exacerbated by food and water insecurity [13,14,15,16]. Disparities in healthcare access, particularly between urban and rural areas, further complicate the management practices.

This study examined how maternal, child, and household factors influence diarrhea management in Ecuador using ENSANUT 2018 data. We assessed medical care attendance, consultation with health professionals, and fluid and dietary management during episodes, considering independent factors, such as urban or rural residence, household size, socioeconomic status, and maternal education. Understanding these determinants can inform targeted public health interventions to improve child-health outcomes in Ecuador.

## 2. Materials and Methods

### 2.1. Study Size

The survey employed a probabilistic, multistage, stratified sampling design to ensure national representativeness. The original ENSANUT sample included 20,510 women of reproductive age (12–49 years) and children under five years old. For this study, the analytic sample was restricted to children under five who had experienced diarrhea in the past two weeks and had complete data for the variables of interest, resulting in a final sample size of 1749. This sample size was well above the typical thresholds for representativeness in population studies, ensuring that the findings reliably reflected the target population. Combined with the probabilistic sampling methodology of ENSANUT, this guarantees the representativeness of the analytical subset.

**Figure 1:**
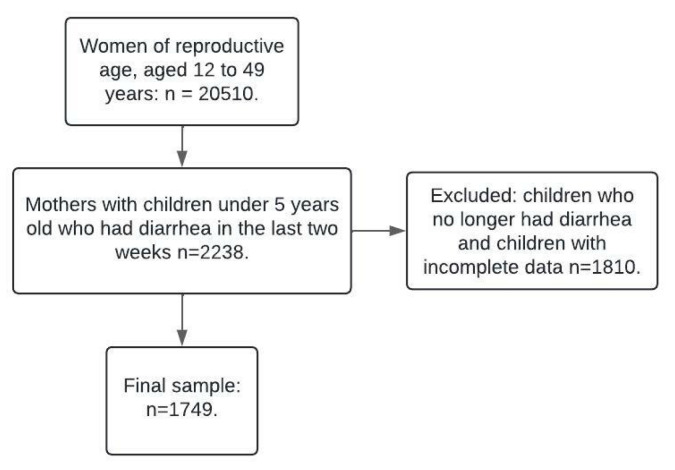
Study size.

Figure 1 shows the exclusion criteria used to determine the study sample. Starting with a population of 20,510, the analytical sample was limited to children under five years of age who had experienced diarrhea in the previous two weeks and had complete data on the variables of interest, resulting in a final sample size of 1749.

### 2.2. Study Design and Scope

This study involved secondary research based on data from the National Health and Nutrition Survey (ENSANUT) 2018 of Ecuador. Data from a nationally representative sample were used to examine the relationships between variables of interest. The study design was cross-sectional, using data-cleaning techniques and an assessment of associations between independent and dependent variables. Different covariates were considered potential confounders, and statistical models were adjusted to minimize bias. Participant selection and data collection were performed by the ENSANUT team following internationally standardized procedures, which ensured the validity and reliability of the data used in this study [17].

### 2.3. Variables and Measurement

This study analyzed a range of dependent and independent variables to assess factors influencing maternal management of childhood diarrhea in Ecuador. The dependent variables included actions taken to manage the illness, such as seeking medical care, attending health professionals, changes in fluid intake, dietary modifications, and reduction in solid food intake. Independent variables were categorized at the household, child, and maternal levels, covering aspects such as household location, sanitation, income, child’s age and dehydration level, and maternal education and marital status. Detailed descriptions of all the variables, including coding and survey question references, are provided in Supplementary Table S1.

### 2.4. Statistical Modeling

Descriptive and inferential statistical methods were used for the analysis. Descriptive statistics summarized the data and presented frequencies and percentages for categorical variables. The chi-square test was used to examine associations between variables, medical care, and professional healthcare. Logistic regression models were used to determine factors influencing the maternal management of acute diarrhea in children, reporting odds ratios (ORs) with 95% confidence intervals (CIs) to quantify the strength of associations. Statistical significance was established at the 5% level (*p* < 0.05), and *p*-values between 0.05 and 0.1 were considered marginally significant. All analyses were performed using Stata 18.

## 3. Results

Table 1 presents an excerpt of the descriptive analysis from Supplementary Tables S2 and S3, which includes a comprehensive overview of all the study variables. Among the 1749 children, 90.1% received some form of healthcare for acute diarrheal disease. Urban areas had a higher rate of healthcare attendance (92.2%) compared to rural areas (88.4%), with this difference being statistically significant (*p* = 0.008). Poverty status was not significantly associated with attendance (*p* = 0.368), with similar attendance rates observed across the non-poor (89.3%), poor (90.9%), and extremely poor (92.0%) groups. Children aged 0–11 months had an attendance rate of 86.4%, though differences across age groups were not statistically significant (*p* = 0.102).

There was a significant association between dehydration status and healthcare attendance (*p* < 0.001), with children showing no dehydration having the lowest attendance (76.9%) compared to those with mild (87.6%) or severe dehydration (94.1%). Maternal education was also associated with attendance (*p* = 0.003), with lower rates among children of mothers with no education or literacy-center attendance (71.4%) compared to those with basic (91.3%) or higher education (86.4%). No significant differences were observed by ethnic group (*p* = 0.234), although attendance was slightly lower among Afro-descendant children (87.4%) compared to Indigenous (91.6%) and Mixed ethnicity (90.4%).

Additional variables such as fluid treatment, dietary changes, maternal age, marital status, mobile phone ownership, and water source are included in Supplementary Tables S2 and S3, along with their respective Chi-square test results.

Table 2 presents the determinants of water management approaches for diarrheal disease, using logistic regression models with odds ratios to assess the impact of various child, maternal, and household characteristics on these approaches.

The first model examined the receipt of medical assistance for acute diarrheal illnesses. Households with 4–6 members (OR = 1.584; *p* < 0.05) and those with 7–9 members (OR = 2.006; *p* < 0.05) had significantly higher odds of receiving assistance than the reference group of 2–3 members.

In the second model, the receipt of assistance from healthcare professionals was analyzed. Children aged 19–23 months were significantly more likely to receive professional medical care than those in the younger age group (OR = 0.479, *p* < 0.01).

The third model assessed the likelihood of increased fluid intake as part of diarrhea management. The results showed significant influences in the 12–18 months age group (OR = 1.859, *p* < 0.01) and the 19–23 months age group (OR = 2.182, *p* < 0.01), indicating a higher probability of increased fluid consumption as part of the care regimen for these children.

The fourth model evaluated the likelihood of dietary change. Living in a rural area was associated with a higher probability of dietary changes (OR = 1.260, *p* < 0.1). Children aged 12–18 months had significantly higher odds of experiencing dietary change (OR = 3.016, *p* < 0.01). Additionally, children with mild dehydration had substantially higher odds of dietary change as part of their care (OR = 3.448, *p* < 0.01).

The fifth model focused on the reduction in solid foods. Children from rural areas were more likely to have a reduced intake of solid foods (OR = 1.377, *p* < 0.1). Larger household sizes, specifically those with 7–9 members, were significantly associated with higher odds of reducing solid foods (OR = 2.033, *p* < 0.05). Children aged 48–59 months were less likely to have a reduced intake of solid foods (OR = 0.158, *p* < 0.01).

## 4. Discussion

This study examined how maternal, child, and household factors influence childhood diarrhea management in Ecuador. Among 1749 children, 90.1% received healthcare, with slightly higher rates in urban areas. Poverty status did not significantly affect healthcare access, likely due to Ecuador’s public health initiatives, including mobile health teams and expanded primary-care services [9,18]. Maternal education, household size, and dehydration severity were stronger predictors of care-seeking behavior.

Maternal education is crucial for the management of diarrhea. Mothers with higher education levels adhered better to recommended practices, such as increased fluid intake and dietary modifications, consistent with global findings [1,19]. However, inadequate management among less-educated mothers highlights the need for targeted health literacy programs.

Household size also influences healthcare utilization. Children from larger households (4–6 and 7–9 members) had higher odds of receiving medical care, likely due to family support, but were also more likely to experience dietary restrictions influenced by cultural beliefs [1,2,4,5,6,7,19,20,21]. Addressing these misconceptions through culturally sensitive health education is therefore essential.

Children aged 19–23 months are more likely to receive medical care than younger infants, likely due to increased pathogen exposure as mobility rises [21,22,23]. In contrast, younger infants (0–11 months) had lower healthcare attendance, possibly because of caregiver underestimation of severity or reliance on home remedies. Ethnicity also plays a role, with children of Mixed-ethnicity mothers receiving more medical care [24,25].

Water access was a key determinant. Children in households with improved water sources had higher rates of fluid treatment and dietary change. However, persistent water insecurity continues to challenge rural and indigenous communities where waterborne diseases remain prevalent [15,16]. Addressing these disparities requires infrastructure investments and education on safe water practices [4,5,6,7,19,23,26,27,28,29,30].

Although this study offers important insights into the factors influencing childhood diarrhea management in Ecuador, several limitations must be considered. The data used, while nationally representative, were collected in 2018 and may not reflect current conditions or healthcare practices. However, since the study focuses on the relationships between variables rather than prevalence estimates, the findings remain relevant. Additionally, as a cross-sectional analysis, the study cannot establish causal relationships. The associations identified may be influenced by unmeasured confounding variables or contextual factors not captured in the dataset.

Other limitations include potential response bias and the exclusion of non-measurable elements such as cultural beliefs and quality of care, which may play significant roles in diarrhea management. Reliance on self-reported data may also affect the accuracy of information on the frequency and handling of diarrhea cases. Furthermore, changes in health practices following the COVID-19 pandemic could affect the current applicability of the findings. Despite these constraints, the study contributes meaningful evidence to inform future research and targeted public health interventions.

Despite these limitations, the study’s findings offer actionable insights that can inform policy and guide targeted interventions to improve child-health outcomes in Ecuador. In particular, the results highlight the need for comprehensive public health strategies that address the social determinants of diarrhea management. These strategies should prioritize maternal education, culturally appropriate interventions, and improvements in water security. Expanding maternal health literacy programs, particularly for less-educated mothers, is essential for improving adherence to recommended practices. Such programs should emphasize the recognition of dehydration symptoms, the correct use of Oral Rehydration Salts (ORS), and the importance of continued feeding during illness. To ensure accessibility, public health messaging can be delivered through visual aids, community radio programs, and mobile health (mHealth) platforms.

Culturally tailored interventions should integrate community health workers (CHWs) trained to provide education in indigenous languages [31]. Collaborating with traditional leaders can help counter harmful misconceptions, such as restricting fluid intake during diarrhea episodes [32]. Additionally, bilingual health materials and incentivizing maternal participation in health programs can enhance their effectiveness [33,34].

Improving water security remains a priority, particularly in rural and indigenous communities where inadequate sanitation exacerbates diarrheal disease. Expanding access to piped water, well construction, and water-purification programs can mitigate waterborne disease transmission [35,36,37]. Community-based initiatives should promote household-level water treatment, including subsidized filters, chlorine tablets, and hygiene education [38,39,40].

Strengthening primary healthcare services is necessary. Expanding mobile health teams, particularly in remote areas, could improve the early detection and management of diarrheal disease [41]. Integrating diarrhea treatment training into routine antenatal and postnatal care can equip new mothers with the knowledge to effectively manage childhood illnesses [42].

## 5. Conclusions

The management of childhood diarrhea in Ecuador is shaped by maternal education, household size, child’s age, ethnicity, and water access, with poverty playing a lesser role because of Ecuador’s public healthcare system. While most children receive some form of care, disparities persist, particularly among less educated mothers and communities with limited access to safe water. Targeted interventions that improve maternal health literacy, address cultural barriers, enhance water security, and strengthen healthcare services are essential to improve child-health outcomes and reduce the burden of diarrheal disease in Ecuador.

## Figures and Tables

**Table 1:** Summary of descriptive statistics and bivariate analysis for healthcare variables and covariates (excerpt).

Variable	Total (%)	No Healthcare Attendance (9.9%)	Healthcare Attendance (90.1%)	*p*-Value
Household-level variables				
Rural/Urban				
Rural	976 (55.8%)	113 (11.6% of Rural)	863 (88.4% of Rural)	0.008
Urban	773 (44.2%)	60 (7.8% of Urban)	713 (92.2% of Urban)	
Poverty Classification				
Non-poor	1029 (58.8%)	110 (10.7% of Non-poor)	919 (89.3% of Non-poor)	0.368
Poor	483 (27.6%)	44 (9.1% of Poor)	439 (90.9% of Poor)	
Extremely Poor	237 (13.6%)	19 (8.0% of Extremely Poor)	218 (92.0% of Extremely Poor)	
Sanitary Facilities				
With Facilities	1596 (91.3%)	164 (10.3% of With Facilities)	1432 (89.7% of With Facilities)	0.082
Without Facilities	153 (8.7%)	9 (5.9% of Without Facilities)	144 (94.1% of Without Facilities)	
Water Source				
Improved Water	1572 (89.9%)	164 (10.4% of Improved Water)	1408 (89.6% of Improved Water)	0.024
Unimproved Water	177 (10.1%)	9 (5.1% of Unimproved Water)	168 (94.9% of Unimproved Water)	
Child-level variables				
Dehydration Indicator				
No Dehydration	212 (12.1%)	49 (23.1% of No Dehydration)	163 (76.9% of No Dehydration)	<0.001
Mild Dehydration	509 (29.1%)	63 (12.4% of Mild Dehydration)	446 (87.6% of Mild Dehydration)	
Severe Dehydration	1028 (58.8%)	61 (5.9% of Severe Dehydration)	967 (94.1% of Severe Dehydration)	
Age in Months				
0–11 Months	346 (19.8%)	47 (13.6% of 0–11 Months)	299 (86.4% of 0–11 Months)	0.102
12–18 Months	390 (22.3%)	40 (10.3% of 12–18 Months)	350 (89.7% of 12–18 Months)	
Maternal-level variables				
Educational Level				
None or Literacy Center	21 (1.2%)	6 (28.6% of None)	15 (71.4% of None)	0.003
Basic Education	668 (38.2%)	58 (8.7% of Basic Education)	610 (91.3% of Basic Education)	
Higher Education	279 (16.0%)	38 (13.6% of Higher Education)	241 (86.4% of Higher Education)	
Race				
Mixed	1264 (72.3%)	122 (9.6% of Mixed)	1142 (90.4% of Mixed)	0.234
Indigenous	296 (16.9%)	25 (8.4% of Indigenous)	271 (91.6% of Indigenous)	
Afro	103 (5.9%)	13 (12.6% of Afro)	90 (87.4% of Afro)	

**Table 2:** Logistic regression analysis of factors associated with healthcare service attendance and childcare practices among mothers of children with diarrhea.

	Outcomes				
Variables	Received Healthcare Attendance	Received Healthcare Professional Attendance	Giving More Liquid	Change in Diet	Decreased Intake of Solids
Rural (Ref = Urban)	1.345	0.926	1.080	1.260 *	1.377 *
	(0.276)	(0.120)	(0.148)	(0.159)	(0.268)
Number of persons in the household (Ref = 2–3 people)					
4–6 people	1.584 **	0.891	0.965	1.113	1.178
	(0.337)	(0.131)	(0.148)	(0.155)	(0.292)
7–9 people	2.006 **	1.116	1.259	1.048	2.033 **
	(0.617)	(0.224)	(0.263)	(0.207)	(0.625)
10+ people	1.355	0.997	0.851	0.876	1.352
	(0.547)	(0.300)	(0.253)	(0.248)	(0.605)
Poverty classification (Ref = not poor)					
Poor	0.874	1.050	0.832	0.918	0.768
	(0.195)	(0.147)	(0.117)	(0.123)	(0.171)
Extremely poor	0.799	1.232	0.978	1.259	0.530 *
	(0.280)	(0.274)	(0.231)	(0.276)	(0.194)
Bad hand washing (Ref = good hand washing)	1.079	0.888	0.751	0.688 *	1.192
	(0.382)	(0.176)	(0.155)	(0.142)	(0.360)
Sanitary facilities (Ref = no sanitary facilities)	0.604	1.549 *	0.898	1.471	0.832
	(0.265)	(0.386)	(0.231)	(0.365)	(0.332)
Household income (Ref = no income)					
$501–$1000	0.929	0.980	1.034	0.837	0.999
	(0.207)	(0.136)	(0.148)	(0.110)	(0.218)
$1001–$1500	0.708	1.106	1.094	1.146	0.901
	(0.204)	(0.237)	(0.235)	(0.236)	(0.292)
$1501–$2000	1.279	0.839	1.535	1.517	1.396
	(0.600)	(0.228)	(0.478)	(0.428)	(0.556)
$2001–$2500	0.560	0.873	0.833	1.208	1.791
	(0.266)	(0.314)	(0.296)	(0.404)	(0.750)
$2501–$3000	0.626	1.160	1.169	1.272	0.448
	(0.401)	(0.579)	(0.594)	(0.617)	(0.470)
$3001–$4000	0.490	0.834	1.724	0.948	0.650
	(0.248)	(0.405)	(0.835)	(0.390)	(0.492)
$4000+	1.137	0.962	1.834	0.753	0.465
	(0.936)	(0.452)	(1.002)	(0.367)	(0.506)
Age in months (Ref = 0–11)					
12–18 months	1.314	0.786	1.859 ***	3.016 ***	0.369 ***
	(0.319)	(0.135)	(0.301)	(0.491)	(0.0902)
19–23 months	2.366 **	0.479 ***	2.182 ***	4.359 ***	0.479 **
	(0.796)	(0.0952)	(0.450)	(0.841)	(0.141)
24–30 months	1.332	0.651 **	2.125 ***	3.008 ***	0.461 ***
	(0.379)	(0.127)	(0.415)	(0.575)	(0.137)
31–35 months	1.393	0.587 **	2.614 ***	2.646 ***	0.321 ***
	(0.507)	(0.131)	(0.624)	(0.575)	(0.124)
36–42 months	2.183 *	0.685	2.258 ***	3.358 ***	0.365 ***
	(0.904)	(0.158)	(0.527)	(0.762)	(0.130)
43–47 months	0.886	0.875	2.692 ***	3.027 ***	0.442 **
	(0.330)	(0.238)	(0.799)	(0.800)	(0.177)
48–59 months	1.217	0.634 **	2.964 ***	3.911 ***	0.158 ***
	(0.359)	(0.132)	(0.628)	(0.785)	(0.0700)
Order of child (Ref = first son)					
Second child	0.779	0.989	0.685 *	1.187	1.169
	(0.251)	(0.192)	(0.139)	(0.237)	(0.409)
Third child		0.628	3.017	0.883	2.176
		(0.385)	(3.615)	(0.542)	(2.006)
Female (Ref = Male)	1.145	0.940	1.019	1.088	1.089
	(0.197)	(0.101)	(0.112)	(0.113)	(0.185)
Dehydrated (Ref = Hydrated)					
Mild dehydration	1.980 ***	1.379 *	3.448 ***	1.761 ***	0.767
	(0.439)	(0.268)	(0.631)	(0.323)	(0.216)
Severe dehydration	4.227 ***	2.039 ***	3.897 ***	3.248 ***	0.922
	(0.921)	(0.376)	(0.671)	(0.563)	(0.237)
Persistent diarrhea (Ref = Acute diarrhea)	0.710		0.388	1.851	0.921
	(0.821)		(0.258)	(1.647)	(1.101)
Race (Ref = Mestizo)					
Indigenous	0.726	0.862	0.748	0.710 **	0.727
	(0.256)	(0.147)	(0.132)	(0.121)	(0.212)
Afro	0.709	0.686	0.772	0.639 **	0.525
	(0.242)	(0.159)	(0.175)	(0.145)	(0.238)
Others	0.620	0.589 **	1.177	0.495 ***	0.730
	(0.213)	(0.158)	(0.330)	(0.126)	(0.312)
Without education (Ref = without education)					
Basic education	4.077 **	0.460	0.640	0.406 *	1.768
	(2.313)	(0.290)	(0.347)	(0.209)	(1.886)
Middle-/high-school education	5.007 ***	0.591	0.870	0.500	1.589
	(2.876)	(0.374)	(0.476)	(0.258)	(1.715)
Higher education	3.275 **	0.749	0.952	0.575	1.383
	(1.953)	(0.486)	(0.541)	(0.307)	(1.544)
Marital status (Ref = Married/United)					
Separated	1.021	1.017	1.346	0.750	1.154
	(0.291)	(0.193)	(0.278)	(0.141)	(0.351)
Single	1.249	0.834	0.835	0.690 **	1.280
	(0.331)	(0.136)	(0.139)	(0.110)	(0.306)
Age in years (Ref = 12–17 years)					
18–19 years	0.697	0.713	0.868	0.498 **	1.077
	(0.366)	(0.263)	(0.304)	(0.175)	(0.527)
20–49 years	1.235	0.789	1.126	1.011	0.667
	(0.617)	(0.261)	(0.351)	(0.322)	(0.311)
Cellphone (Ref = without cellphone)	1.335	1.179	1.058	0.915	1.965 ***
	(0.291)	(0.161)	(0.147)	(0.121)	(0.468)
Improved Water (Ref = unimproved water)	0.496 *	1.223	1.432 *	1.138	0.571 *
	(0.210)	(0.257)	(0.299)	(0.242)	(0.183)
Constant	1.037	1.733	0.380	0.298 *	0.191
	(1.023)	(1.372)	(0.263)	(0.201)	(0.260)
Observations	1740	1576	1749	1749	1749

Robust standard errors are indicated in parentheses. *** *p* < 0.01, ** *p* < 0.05, * *p* < 0.1.

## Data Availability

The data used in this study were obtained from the 2018 National Health and Nutrition Survey of Ecuador (Ensanut) and can be freely accessed from the following website: https://www.ecuadorencifras.gob.ec/salud-salud-reproductiva-y-nutricion/ (accessed on 23 June 2024).

## Supplementary Materials

The following supporting information can be downloaded at: https://www.mdpi.com/article/10.3390/children12040473/s1, Table S1: Models Variables; Table S2: Descriptive statistics and bivariate analysis for health care variables and covariates (all of them). Table S3: Summary of descriptive statistics and bivariate analysis of health care variables and covariates.

## References

[1] Momoh F.E., Olufela O.E., Adejimi A.A., Roberts A.A., Oluwole E.O., Ayankogbe O.O., Onajole A.T. (2022). Mothers’ Knowledge, Attitude and Home Management of Diarrhoea among Children under Five Years Old in Lagos, Nigeria. Afr. J. Prim. Health Care Fam. Med..

[2] Diouf K., Tabatabai P., Rudolph J., Marx M. (2014). Diarrhoea Prevalence in Children under Five Years of Age in Rural Burundi: An Assessment of Social and Behavioural Factors at the Household Level. Glob. Health Action.

[3] Diarrheal Diseases. https://www.who.int/es/news-room/fact-sheets/detail/diarrhoeal-disease.

[4] Shewangizaw B., Mekonen M., Fako T., Hoyiso D., Borie Y.A., Yeheyis T., Kassahun G. (2023). Knowledge and Attitude on Home-Based Management of Diarrheal Disease among Mothers/Caregivers of under-Five Children at a Tertiary Hospital in Ethiopia. Pan Afr. Med. J..

[5] Workie H.M., Sharifabdilahi A.S., Addis E.M. (2018). Mothers’ Knowledge, Attitude and Practice towards the Prevention and Home-Based Management of Diarrheal Disease among under-Five Children in Diredawa, Eastern Ethiopia, 2016: A Cross-Sectional Study. BMC Pediatr..

[6] Terefe G., Murugan R., Bedada T., Bacha G., Bekele G. (2022). Home-Based Management Practice of Diarrhea in under 5 Years Old Children and Associated Factors among Caregivers in Ginchi Town, Oromia Region, West Ethiopia. SAGE Open Med..

[7] Merga N., Alemayehu T. (2015). Knowledge, Perception, and Management Skills of Mothers with under-Five Children about Diarrhoeal Disease in Indigenous and Resettlement Communities in Assosa District, Western Ethiopia. J. Health Popul. Nutr..

[8] Sánchez X., Calderón N., Solis O., Jimbo-Sotomayor R. (2023). Antibiotic Prescription Patterns in Children Under 5 Years of Age With Acute Diarrhea in Quito-Ecuador. J. Prim. Care Community Health.

[9] Galárraga O., Quijano-Ruiz A., Faytong-Haro M. (2024). The Effects of Mobile Primary Health Teams: Evidence from the Médico Del Barrio Strategy in Ecuador. World Dev..

[10] Mora F.X., Avilés-Reyes R.X., Guerrero-Latorre L., Fernández-Moreira E. (2016). Atypical Enteropathogenic Escherichia Coli (aEPEC) in Children under Five Years Old with Diarrhea in Quito (Ecuador). Int. Microbiol..

[11] Montero L., Smith S.M., Jesser K.J., Paez M., Ortega E., Peña-Gonzalez A., Soto-Girón M.J., Hatt J.K., Sánchez X., Puebla E. (2023). Distribution of Escherichia Coli Pathotypes along an Urban–Rural Gradient in Ecuador. Am. J. Trop. Med. Hyg..

[12] Jacobsen K.H., Ribeiro P.S., Quist B.K., Rydbeck B.V. (2007). Prevalence of Intestinal Parasites in Young Quichua Children in the Highlands of Rural Ecuador. J. Health Popul. Nutr..

[13] Román-Zambrano V.V., González-Hernández A. (2023). Malnutrition and Its Influence on Morbidity in Children Under Five Years of Age, Crucita Parish, Manabí. MQRInvestigar [Internet]. https://www.investigarmqr.com/ojs/index.php/mqr/article/view/766.

[14] Vera A.A.Z., Vargas D.M.Z., Nieto L.C.M. (2024). Nivel de Desnutrición a Partir de Las Medidas Antropométricas En Niños Menores de 5 Años. Polo Conoc..

[15] Ortiz-Prado E., Simbaña-Rivera K., Cevallos G., Gómez-Barreno L., Cevallos D., Lister A., Fernandez-Naranjo R., Ríos-Touma B., Vásconez-González J., Izquierdo-Condoy J.S. (2022). Waterborne Diseases and Ethnic-Related Disparities: A 10 Years Nationwide Mortality and Burden of Disease Analysis from Ecuador. Front. Public Health.

[16] Chakraborty R., Armijos R.X., Beidelman E.T., Rosenberg M., Margaret Weigel M. (2024). Household Food and Water Insecurity and Its Association with Diarrhoea, Respiratory Illness, and Stunting in Ecuadorian Children under 5 Years. Matern. Child. Nutr..

[17] Ecuador—National Health and Nutrition Survey 2018—General Information. https://anda.inec.gob.ec/anda/index.php/catalog/891.

[18] Lucio R., Villacrés N., Henríquez R. (2011). Sistema de salud de Ecuador. Salud Pública de México.

[19] Kaçan C.Y., Palloş A., Özkaya G. (2022). Examining Knowledge and Traditional Practices of Mothers with Children under Five in Turkey on Diarrhoea According to Education Levels. Ann. Med..

[20] Thiam S., Sy I., Schindler C., Niang-Diène A., Faye O., Utzinger J., Cissé G. (2019). Knowledge and Practices of Mothers and Caregivers on Diarrhoeal Management among under 5-Year-Old Children in a Medium-Size Town of Senegal. Acta Trop..

[21] Rahman M.M., Saima U., Goni M.A. (2015). Impact of Maternal Household Decision-Making Autonomy on Child Nutritional Status in Bangladesh. Asia Pac. J. Public Health.

[22] Islam M.S., Chowdhury M.R.K., Bornee F.A., Chowdhury H.A., Billah B., Kader M., Rashid M. (2023). Prevalence and Determinants of Diarrhea, Fever, and Coexistence of Diarrhea and Fever in Children under-Five in Bangladesh. Children.

[23] Simelane M., Vermaak K. (2023). A Multilevel Analysis of Individual, Household and Community Level Predictors of Child Diarrhea in Eswatini. J. Public Health Afr..

[24] Jones E., Lattof S.R., Coast E. (2017). Interventions to Provide Culturally-Appropriate Maternity Care Services: Factors Affecting Implementation. BMC Pregnancy Childbirth.

[25] Yuan B., Målqvist M., Trygg N., Qian X., Ng N., Thomsen S. (2014). What Interventions Are Effective on Reducing Inequalities in Maternal and Child Health in Low-and Middle-Income Settings? A Systematic Review. BMC Public Health.

[26] Claudine U., Kim J.Y., Kim E.-M., Yong T.-S. (2021). Association between Sociodemographic Factors and Diarrhea in Children under 5 Years in Rwanda. Korean J. Parasitol..

[27] Wasihun A.G., Dejene T.A., Teferi M., Marugán J., Negash L., Yemane D., McGuigan K.G. (2018). Risk Factors for Diarrhoea and Malnutrition among Children under the Age of 5 Years in the Tigray Region of Northern Ethiopia. PLoS ONE.

[28] Wolf J., Hubbard S., Brauer M., Ambelu A., Arnold B.F., Bain R., Bauza V., Brown J., Caruso B.A., Clasen T. (2022). Effectiveness of Interventions to Improve Drinking Water, Sanitation, and Handwashing with Soap on Risk of Diarrhoeal Disease in Children in Low-Income and Middle-Income Settings: A Systematic Review and Meta-Analysis. Lancet.

[29] Adane M., Mengistie B., Medhin G., Kloos H., Mulat W. (2017). Piped Water Supply Interruptions and Acute Diarrhea among Under-Five Children in Addis Ababa Slums, Ethiopia: A Matched Case-Control Study. PLoS ONE.

[30] Bhavnani D., Goldstick J.E., Cevallos W., Trueba G., Eisenberg J.N. (2014). Impact of Rainfall on Diarrheal Disease Risk Associated with Unimproved Water and Sanitation. Am. J. Trop. Med. Hyg..

[31] Nyamathi A., Jackson D., Carter B., Hayter M. (2012). Creating Culturally Relevant and Sustainable Research Strategies to Meet the Needs of Vulnerable Populations. Contemp. Nurse.

[32] Martínez H., Habicht J.-P. (1996). A Programme to Develop Culturally and Medically Sound Home Fluid Management of Children with Acute Diarrhoea. Food Nutr. Bull..

[33] McCarty D.B., Sierra-Arevalo L., Caldwell Ashur A.-C., White J.T., Villa Torres L. (2024). Spanish Translation and Cultural Adaptations of Physical Therapy Parent Educational Materials for Use in Neonatal Intensive Care. Patient Prefer. Adherence.

[34] Miller L.R. (2022). The Use of an Incentive to Improve Breastfeeding Outcomes: The Effectiveness of Offering a Free Family YMCA Membership to Increase Support Group Participation. J. Hum. Lact..

[35] Nandi A., Megiddo I., Ashok A., Verma A., Laxminarayan R. (2017). Reduced Burden of Childhood Diarrheal Diseases through Increased Access to Water and Sanitation in India: A Modeling Analysis. Soc. Sci. Med..

[36] Torres-Slimming P.A., Carcamo C.P., Wright C.J., Lancha G., Zavaleta-Cortijo C., King N., Ford J.D., Garcia P.J., Harper S.L. (2023). Diarrheal Disease and Associations with Water Access and Sanitation in Indigenous Shawi Children along the Armanayacu River Basin in Peru. Rural. Remote Health.

[37] Gallandat K., Macdougall A., Jeandron A., Mufitini Saidi J., Bashige Rumedeka B., Malembaka E.B., Azman A.S., Bompangue D., Cousens S., Allen E. (2024). Improved Water Supply Infrastructure to Reduce Acute Diarrhoeal Diseases and Cholera in Uvira, Democratic Republic of the Congo: Results and Lessons Learned from a Pragmatic Trial. PLoS Neglected Trop. Dis..

[38] Bick R., Talboys S., Vanderslice J., Stringer K. (2014). Evaluation of a Village-Level Safe Water Treatment and Storage Intervention in Bassi Pathana, India. Ann. Glob. Health.

[39] Clasen T., Garcia Parra G., Boisson S., Collin S. (2005). Household-Based Ceramic Water Filters for the Prevention of Diarrhea: A Randomized, Controlled Trial of a Pilot Program in Colombia. Am. J. Trop. Med. Hyg..

[40] Freeman M.C., Trinies V., Boisson S., Mak G., Clasen T. (2012). Promoting Household Water Treatment through Women’s Self Help Groups in Rural India: Assessing Impact on Drinking Water Quality and Equity. PLoS ONE.

[41] Bhutta Z.A. (2020). Reaching the Unreached; Mobile Health Teams in Conflict Settings. Arch. Dis. Child..

[42] Abua U.J., Igbudu T.J., Egwuda L., Yaakugh G.J. (2020). Impact of Training of Primary Healthcare Workers on Integrated Community Case Management of Childhood Illnesses in North-West District of Benue State, Nigeria. J. Pharm. Bioresour..

