# Supplementary material for: Influences of Maternal, Child, and Household Factors on Diarrhea Management in Ecuador
Source: Children (Basel). 2025 Apr 7;12(4):473. doi: 10.3390/children12040473 (PMC12025387; doi:10.3390/children12040473)
Supplement: Supplementary file 1 [file children-12-00473-s001.zip › children-3505404-supplementary.pdf]

Supplementary Table S1: Models Variables

Category	Variable	Description	Code	Survey Section and Question
**Dependent Variables**	**Medical care attendance**	Action taken by the mother to relieve her child's illness for those with diarrhea in the past two weeks.	0 = No action taken, 1 = Action taken	Section G, Question 463: Did you take any action to relieve your child's illness in the past two weeks?
	**Attendance to health professionals**	Care received from health professionals (doctor, nurse, or aide) for children who sought care.	0 = No professional care, 1 = Received professional care	Section G, Question 469: Did the child receive care from a doctor, nurse, or nurse's aide for the illness?
	**Giving more fluids**	Changes in the amount of fluids given to the child during diarrhea episodes.	1 = More fluids, 0 = Fewer fluids or same amount of fluids	Section G, Question 470: Did you increase, reduce, or maintain the usual amount of fluids during the episode?
	**Change in diet**	Modification in the child's diet during diarrhea episodes.	0 = No change, 1 = Diet modified	Section G, Question 471: Did you make any changes to the child's diet during the episode?
	**Decreased solid intake**	Reduction in the amount of solid foods given during diarrhea episodes.	0 = No reduction (or no solids), 1 = Reduction in solids	Section G, Question 472: Did you decrease, maintain, or increase the amount of solid

				food given during the episode?
**Household Level**	**Urban or rural**	Household location (urban or rural).	1 = Urban, 0 = Rural	Section A, Questions 1 and 2: Is the household in an urban or rural area?
	**Number of persons in the household**	Total number of household members.	Integer value	Section C, Question 3: How many persons are living in the household?
	**Poverty classification**	Household classification based on the Unsatisfied Basic Needs (UBN) method.	Non-poor = No indicators; Poor = 1 indicator; Extremely poor = ≥2 indicators	Derived from Section C, Multiple Indicators: Electricity, water, overcrowding, schooling, etc.
	**Poor handwashing**	Handwashing practices in the household.	0 = Good practices, 1 = Poor practices	Section 1, Question 36: What implements are used for handwashing (e.g., soap, detergent, none)?
	**Sanitary facilities**	Availability of sanitation facilities in the household.	0 = No facilities, 1 = With facilities	Section 1, Question 13: What type of sanitation facilities does the household have (e.g., latrine, toilet)?
	**Household income**	Total household income, covering all sources.	Income ranges (e.g., 501–1000, 1001–1500)	Section 3, Questions 15–30: What is the household income

				from all sources?
	**Improved water**	Source of drinking water for the household.	1 = Improved water, 0 = Unimproved water<br><br>*Improved water includes piped sources, protected wells and springs, rainwater, and bottled water (with safe alternatives for other uses), while unimproved water includes unprotected sources, surface water, and tanker or cart deliveries.	Section 2, Question 25: What is the main source of drinking water for the household (e.g., bottled, piped, well)?
**Child Level**	**Age in months**	Child's age in months.	Categories: 12–18, 19–23, ..., 48–59 months	Section 2, Question 3: What is the child's age in months?
	**Order of the child**	Birth order of the child within the household.	Categorical values (First, Second, Third)	Section IV, Child Health: What is the order of the child in the family?
	**Male or female**	Child's sex.	1 = Male, 0 = Female	Section 2, Question 3: Is the child male or female?
	**Level of dehydration**	Level of dehydration during diarrhea episodes based on clinical symptoms.	Mild, Moderate, Severe	Section G, Question 262: What symptoms did the child exhibit during diarrhea (e.g., sunken eyes, thirst, lethargy)?
	**Persistent**	Duration of the	0 = Acute (<2 weeks), 1 =	Section G,

	**diarrhea**	diarrhea episode.	Persistent (≥2 weeks)	Question 258: How long did the diarrhea episode last?
**Maternal Level**	**Ethnicity**	Mother's cultural or ethnic background.	Mestizo, Indigenous, Afro, Other	Section 2, Question 9: What is the mother's cultural or ethnic background?
	**Level of education**	Mother's highest educational attainment.	None, Basic, Secondary, Higher education	Section 2, Question 19: What is the highest level of education attained by the mother?
	**Marital status**	Mother's marital or conjugal status.	Married/United, Separated, Single	Section 2, Question 16: What is the mother's current marital status (e.g., married, separated)?
	**Age in years**	Mother's age.	12–17, 18–19, 20–49 years	Section 2, Question 3: What is the mother's age?
	**Cell phone ownership**	Mother's ownership of an activated cell phone.	1 = Owns a cell phone, 0 = Does not own a cell phone	Section 2, Question 23: Does the mother own a cell phone?

Supplementary Table S2: Descriptive statistics and bivariate analysis for health care variables and covariates (all of them).

	Total	Received health care attendance			Received healthcare professional treatment		
		**No**	**Yes**	Test	No	Yes	Test
**VARIABLE**	1,749 (100.0%)	173 (9.9%)	1,576 (90.1%)		628 (39.8%)	948 (60.2%)	
**Household-level variables**							
**Rural/Urban**							
Rural	976 (55.8%)	113 (65.3%)	863 (54.8%)	0.008	317 (50.5%)	546 (57.6%)	0.005
Urban	773 (44.2%)	60 (34.7%)	713 (45.2%)		311 (49.5%)	402 (42.4%)	
**Number of people in the household**							
1-3 people	359 (20.5%)	46 (26.6%)	313 (19.9%)	0.101	120 (19.1%)	193 (20.4%)	0.670
4-6 people	1,009 (57.7%)	92 (53.2%)	917 (58.2%)		375 (59.7%)	542 (57.2%)	
7-9 ppeople	288 (16.5%)	23 (13.3%)	265 (16.8%)		99 (15.8%)	166 (17.5%)	
10+ people	93 (5.3%)	12 (6.9%)	81 (5.1%)		34 (5.4%)	47 (5.0%)	
**Poverty classification**							
Not poor	1,029 (58.8%)	110 (63.6%)	919 (58.3%)	0.368	351 (55.9%)	568 (59.9%)	0.196
Poor	483 (27.6%)	44 (25.4%)	439 (27.9%)		180 (28.7%)	259 (27.3%)	
Extremely poor	237 (13.6%)	19 (11.0%)	218 (13.8%)		97 (15.4%)	121 (12.8%)	
**Bad hand washing**	0.098 (0.298)	0.069 (0.255)	0.102 (0.302)	0.178	0.119 (0.325)	0.090 (0.286)	0.055
Sanitary facilities	0.913 (0.283)	0.948 (0.223)	0.909 (0.288)	0.082	0.882 (0.323)	0.926 (0.262)	0.003
**Household income**							
Less than $500	857 (49.0%)	76 (43.9%)	781 (49.6%)	0.365	328 (52.2%)	453 (47.8%)	0.666
$501- $1000	505 (28.9%)	50 (28.9%)	455 (28.9%)		176 (28.0%)	279 (29.4%)	
$1001- $1500	172 (9.8%)	22 (12.7%)	150 (9.5%)		51 (8.1%)	99 (10.4%)	
$1501- $2000	93 (5.3%)	7 (4.0%)	86 (5.5%)		35 (5.6%)	51 (5.4%)	

$2001- $2500	48 (2.7%)	7 (4.0%)	41 (2.6%)		16 (2.5%)	25 (2.6%)	
$2501- $3000	24 (1.4%)	4 (2.3%)	20 (1.3%)		6 (1.0%)	14 (1.5%)	
$3001- $4000	28 (1.6%)	5 (2.9%)	23 (1.5%)		9 (1.4%)	14 (1.5%)	
$ 4000+	22 (1.3%)	2 (1.2%)	20 (1.3%)		7 (1.1%)	13 (1.4%)	
**Water status**							
Unimproved	177 (10.1%)	9 (5.2%)	168 (10.7%)	0.024	82 (13.1%)	86 (9.1%)	0.012
Improved	1,572 (89.9%)	164 (94.8%)	1,408 (89.3%)		546 (86.9%)	862 (90.9%)	
**Child-level variables**							
**Age in months**							
0-11 months	346 (19.8%)	47 (27.2%)	299 (19.0%)	0.102	99 (15.8%)	200 (21.1%)	0.024
12-18 months	390 (22.3%)	40 (23.1%)	350 (22.2%)		130 (20.7%)	220 (23.2%)	
19-23 months	200 (11.4%)	12 (6.9%)	188 (11.9%)		92 (14.6%)	96 (10.1%)	
24-30 months	223 (12.8%)	22 (12.7%)	201 (12.8%)		85 (13.5%)	116 (12.2%)	
31-35 months	137 (7.8%)	11 (6.4%)	126 (8.0%)		56 (8.9%)	70 (7.4%)	
36-42 months	146 (8.3%)	9 (5.2%)	137 (8.7%)		55 (8.8%)	82 (8.6%)	
43-47 months	93 (5.3%)	11 (6.4%)	82 (5.2%)		29 (4.6%)	53 (5.6%)	
48-59 months	214 (12.2%)	21 (12.1%)	193 (12.2%)		82 (13.1%)	111 (11.7%)	
**Order of child**							
1	1,590 (90.9%)	159 (91.9%)	1,431 (90.8%)	0.588	563 (89.6%)	868 (91.6%)	0.348
2	150 (8.6%)	14 (8.1%)	136 (8.6%)		60 (9.6%)	76 (8.0%)	
3	9 (0.5%)	0 (0.0%)	9 (0.6%)		5 (0.8%)	4 (0.4%)	
**Child Sex**							
Male	919 (52.5%)	95 (54.9%)	824 (52.3%)	0.511	323 (51.4%)	501 (52.8%)	0.582
Female	830 (47.5%)	78 (45.1%)	752 (47.7%)		305 (48.6%)	447 (47.2%)	
**Dehydration indicator**							
No Dehydration	212 (12.1%)	49 (28.3%)	163 (10.3%)	<0.001	82 (13.1%)	81 (8.5%)	0.001
Mild Dehydration	509	63	446		192	254	

	(29.1%)	(36.4%)	(28.3%)		(30.6%)	(26.8%)	
Severe Dehydration	1,028 (58.8%)	61 (35.3%)	967 (61.4%)		354 (56.4%)	613 (64.7%)	
**Type of diarrhea**							
Acute	1,740 (99.5%)	172 (99.4%)	1,568 (99.5%)	0.902	628 (100.0%)	940 (99.2%)	0.021
Persistent	9 (0.5%)	1 (0.6%)	8 (0.5%)		0 (0.0%)	8 (0.8%)	
**Mother-level variables**							
**Race**							
Mixed	1,264 (72.3%)	122 (70.5%)	1,142 (72.5%)	0.234	424 (67.5%)	718 (75.7%)	0.003
Indigenous	296 (16.9%)	25 (14.5%)	271 (17.2%)		124 (19.7%)	147 (15.5%)	
Afro	103 (5.9%)	13 (7.5%)	90 (5.7%)		41 (6.5%)	49 (5.2%)	
Other	86 (4.9%)	13 (7.5%)	73 (4.6%)		39 (6.2%)	34 (3.6%)	
**Educational level**							
None or literacy center	21 (1.2%)	6 (3.5%)	15 (1.0%)	0.003	4 (0.6%)	11 (1.2%)	0.005
Basic education	668 (38.2%)	58 (33.5%)	610 (38.7%)		273 (43.5%)	337 (35.5%)	
Middle/high school education	781 (44.7%)	71 (41.0%)	710 (45.1%)		272 (43.3%)	438 (46.2%)	
Higher education	279 (16.0%)	38 (22.0%)	241 (15.3%)		79 (12.6%)	162 (17.1%)	
**Marital Status**							
Married/United	1,338 (76.5%)	136 (78.6%)	1,202 (76.3%)	0.703	480 (76.4%)	722 (76.2%)	0.836
Separated	173 (9.9%)	17 (9.8%)	156 (9.9%)		59 (9.4%)	97 (10.2%)	
Single	238 (13.6%)	20 (11.6%)	218 (13.8%)		89 (14.2%)	129 (13.6%)	
**Age in years**							
12-17 years	56 (3.2%)	6 (3.5%)	50 (3.2%)	0.072	18 (2.9%)	32 (3.4%)	0.594
18-19 years	160 (9.1%)	24 (13.9%)	136 (8.6%)		59 (9.4%)	77 (8.1%)	
20-49 years	1,533 (87.7%)	143 (82.7%)	1,390 (88.2%)		551 (87.7%)	839 (88.5%)	
**Cellphone ownership**	0.688 (0.464)	0.688 (0.465)	0.688 (0.464)	0.999	0.646 (0.478)	0.715 (0.452)	0.004

Supplementary Table S3. Summary of descriptive statistics and bivariate analysis of health care variables and covariates (continued).

VARIABLE	Treatment: More liquid			Treatment: Change in diet			Treatment: Decreased intake of solids		
**TOTAL**	**No**	**Yes**	**Test**	**No**	**Yes**	Test	No	Yes	Test
	533 (30.5%)	1,216 (69.5%)		790 (45.2%)	959 (54.8%)		1,639 (93.7%)	110 (6.3%)	
**Household-level variables**									
**Rural/Urban**									
Rural	278 (52.2%)	698 (57.4%)	0.042	446 (56.5%)	530 (55.3%)	0.618	918 (56.0%)	58 (52.7%)	0.502
Urban	255 (47.8%)	518 (42.6%)		344 (43.5%)	429 (44.7%)		721 (44.0%)	52 (47.3%)	
**Number of people in the household**									
1-3 people	104 (19.5%)	255 (21.0%)	0.190	170 (21.5%)	189 (19.7%)	0.137	340 (20.7%)	19 (17.3%)	0.017
4-6 people	310 (58.2%)	699 (57.5%)		435 (55.1%)	574 (59.9%)		954 (58.2%)	55 (50.0%)	
7-9 ppeople	82 (15.4%)	206 (16.9%)		135 (17.1%)	153 (16.0%)		258 (15.7%)	30 (27.3%)	
10+ people	37 (6.9%)	56 (4.6%)		50 (6.3%)	43 (4.5%)		87 (5.3%)	6 (5.5%)	
**Poverty classification**									
Not poor	286 (53.7%)	743 (61.1%)	0.012	454 (57.5%)	575 (60.0%)	0.556	963 (58.8%)	66 (60.0%)	0.696
Poor	162 (30.4%)	321 (26.4%)		227 (28.7%)	256 (26.7%)		451 (27.5%)	32 (29.1%)	
Extremely poor	85 (15.9%)	152 (12.5%)		109 (13.8%)	128 (13.3%)		225 (13.7%)	12 (10.9%)	
Bad hand washing	0.128 (0.334)	0.086 (0.280)	0.007	0.115 (0.319)	0.084 (0.278)	0.032	0.095 (0.294)	0.145 (0.354)	0.087
Sanitary facilities	0.895 (0.307)	0.920 (0.271)	0.085	0.900 (0.300)	0.923 (0.267)	0.093	0.912 (0.283)	0.918 (0.275)	0.828
**Household income**									
Less than $500	284 (53.3%)	573 (47.1%)	0.149	390 (49.4%)	467 (48.7%)	0.362	810 (49.4%)	47 (42.7%)	0.570
$501- $1000	150 (28.1%)	355 (29.2%)		243 (30.8%)	262 (27.3%)		469 (28.6%)	36 (32.7%)	
$1001- $1500	49 (9.2%)	123 (10.1%)		74 (9.4%)	98 (10.2%)		162 (9.9%)	10 (9.1%)	
$1501- $2000	19 (3.6%)	74 (6.1%)		32 (4.1%)	61 (6.4%)		83 (5.1%)	10 (9.1%)	
$2001- $2500	15	33 (2.7%)		19	29		44 (2.7%)	4 (3.6%)	

	(2.8%)			(2.4%)	(3.0%)				
$2501- $3000	6 (1.1%)	18 (1.5%)		9 (1.1%)	15 (1.6%)		23 (1.4%)	1 (0.9%)	
$3001- $4000	6 (1.1%)	22 (1.8%)		13 (1.6%)	15 (1.6%)		27 (1.6%)	1 (0.9%)	
$ 4000+	4 (0.8%)	18 (1.5%)		10 (1.3%)	12 (1.3%)		21 (1.3%)	1 (0.9%)	
**Water status**									
Unimproved	75 (14.1%)	102 (8.4%)	<0.001	87 (11.0%)	90 (9.4%)	0.261	163 (9.9%)	14 (12.7%)	0.349
Improved	458 (85.9%)	1,114 (91.6%)		703 (89.0%)	869 (90.6%)		1,476 (90.1%)	96 (87.3%)	
**Child-level variables**									
**Age in months**									
0-11 months	159 (29.8%)	187 (15.4%)	<0.001	239 (30.3%)	107 (11.2%)	<0.001	332 (20.3%)	14 (12.7%)	0.090
12-18 months	119 (22.3%)	271 (22.3%)		167 (21.1%)	223 (23.3%)		365 (22.3%)	25 (22.7%)	
19-23 months	55 (10.3%)	145 (11.9%)		69 (8.7%)	131 (13.7%)		181 (11.0%)	19 (17.3%)	
24-30 months	60 (11.3%)	163 (13.4%)		93 (11.8%)	130 (13.6%)		204 (12.4%)	19 (17.3%)	
31-35 months	32 (6.0%)	105 (8.6%)		58 (7.3%)	79 (8.2%)		129 (7.9%)	8 (7.3%)	
36-42 months	38 (7.1%)	108 (8.9%)		54 (6.8%)	92 (9.6%)		135 (8.2%)	11 (10.0%)	
43-47 months	23 (4.3%)	70 (5.8%)		37 (4.7%)	56 (5.8%)		86 (5.2%)	7 (6.4%)	
48-59 months	47 (8.8%)	167 (13.7%)		73 (9.2%)	141 (14.7%)		207 (12.6%)	7 (6.4%)	
**Order of child**									
1	482 (90.4%)	1,108 (91.1%)	0.334	733 (92.8%)	857 (89.4%)	0.040	1,491 (91.0%)	99 (90.0%)	0.818
2	50 (9.4%)	100 (8.2%)		53 (6.7%)	97 (10.1%)		140 (8.5%)	10 (9.1%)	
3	1 (0.2%)	8 (0.7%)		4 (0.5%)	5 (0.5%)		8 (0.5%)	1 (0.9%)	
**Sex**									
Male	282 (52.9%)	637 (52.4%)	0.840	418 (52.9%)	501 (52.2%)	0.780	860 (52.5%)	59 (53.6%)	0.813
Female	251 (47.1%)	579 (47.6%)		372 (47.1%)	458 (47.8%)		779 (47.5%)	51 (46.4%)	
**Dehydration indicator**									
No Dehydration	118 (22.1%)	94 (7.7%)	<0.001	139 (17.6%)	73 (7.6%)	<0.001	204 (12.4%)	8 (7.3%)	0.155
Mild Dehydration	140 (26.3%)	369 (30.3%)		263 (33.3%)	246 (25.7%)		480 (29.3%)	29 (26.4%)	

Severe Dehydration	275 (51.6%)	753 (61.9%)		388 (49.1%)	640 (66.7%)		955 (58.3%)	73 (66.4%)	
**Type of diarrhea**									
Acute	528 (99.1%)	1,212 (99.7%)	0.101	787 (99.6%)	953 (99.4%)	0.474	1,631 (99.5%)	109 (99.1%)	0.550
Persistent	5 (0.9%)	4 (0.3%)		3 (0.4%)	6 (0.6%)		8 (0.5%)	1 (0.9%)	
**Mother-level variables**									
**Race**									
Mixed	361 (67.7%)	903 (74.3%)	0.013	542 (68.6%)	722 (75.3%)	0.007	1,183 (72.2%)	81 (73.6%)	0.934
Indigenous	111 (20.8%)	185 (15.2%)		143 (18.1%)	153 (16.0%)		277 (16.9%)	19 (17.3%)	
Afro	37 (6.9%)	66 (5.4%)		56 (7.1%)	47 (4.9%)		98 (6.0%)	5 (4.5%)	
Other	24 (4.5%)	62 (5.1%)		49 (6.2%)	37 (3.9%)		81 (4.9%)	5 (4.5%)	
**Educational level**									
None or literacy center	7 (1.3%)	14 (1.2%)	0.003	7 (0.9%)	14 (1.5%)	0.138	20 (1.2%)	1 (0.9%)	0.655
Basic education	235 (44.1%)	433 (35.6%)		314 (39.7%)	354 (36.9%)		624 (38.1%)	44 (40.0%)	
Middle/high school education	224 (42.0%)	557 (45.8%)		358 (45.3%)	423 (44.1%)		729 (44.5%)	52 (47.3%)	
Higher education	67 (12.6%)	212 (17.4%)		111 (14.1%)	168 (17.5%)		266 (16.2%)	13 (11.8%)	
**Marital Status**									
Married/United	414 (77.7%)	924 (76.0%)	0.160	582 (73.7%)	756 (78.8%)	0.035	1,261 (76.9%)	77 (70.0%)	0.173
separated	42 (7.9%)	131 (10.8%)		85 (10.8%)	88 (9.2%)		157 (9.6%)	16 (14.5%)	
Single	77 (14.4%)	161 (13.2%)		123 (15.6%)	115 (12.0%)		221 (13.5%)	17 (15.5%)	
**Age in years**									
12-17 years	23 (4.3%)	33 (2.7%)	0.006	29 (3.7%)	27 (2.8%)	<0.001	48 (2.9%)	8 (7.3%)	0.008
18-19 years	63 (11.8%)	97 (8.0%)		102 (12.9%)	58 (6.0%)		145 (8.8%)	15 (13.6%)	
20-49 years	447 (83.9%)	1,086 (89.3%)		659 (83.4%)	874 (91.1%)		1,446 (88.2%)	87 (79.1%)	
**Cellphone**	0.640 (0.481)	0.709 (0.454)	0.004	0.681 (0.466)	0.693 (0.461)	0.577	0.685 (0.465)	0.736 (0.443)	0.257
